# Distant metastasis risk and patterns of nasopharyngeal carcinoma in the era of IMRT: long-term results and benefits of chemotherapy

**DOI:** 10.18632/oncotarget.4312

**Published:** 2015-05-28

**Authors:** An-Chuan Li, Wei-Wei Xiao, Guan-Zhu Shen, Lin Wang, An-An Xu, Yan-Qing Cao, Shao-Min Huang, Cheng-Guang Lin, Fei Han, Xiao-Wu Deng, Chong Zhao

**Affiliations:** ^1^ Sun Yat-Sen University Cancer Center, State Key Laboratory of Oncology in South China, Collaborative Innovation Center for Cancer Medicine, Guangzhou, China; ^2^ Department of Radiation Oncology, Fujian Medical University Union Hospital, Fuzhou, China; ^3^ Department of Radiation Oncology, Cancer Center of Guangzhou Medical University, Guangzhou, China; ^4^ Department of Radiation Oncology, The First Affiliated Hospital of Clinical Medicine of Guangdong Pharmaceutical University, Guangzhou, China

**Keywords:** nasopharyngeal carcinoma, distant metastasis, intensity-modulated radiotherapy, chemotherapy

## Abstract

Purpose: To report the distant metastasis (DM) risk and patterns for nasopharyngeal carcinoma (NPC) treated with intensity-modulated radiotherapy (IMRT) and to analyze the benefits of chemotherapy based on DM risk.

Materials and Methods: 576 NPC patients were analyzed. The DM rates were calculated using the Kaplan-Meier method, and the log-rank test was used to compare differences. The patients were divided into different risk subclassifications according to DM hazard ratios.

Results: 91 patients developed DM after treatment, with bone as the most common metastatic sites. 82.4% of DMs occurred within 3 years of treatment. Patients were classified as low-risk, intermediate-risk and high-risk, and the corresponding 5-year DM rates were 5.1%, 13.1% and 32.4%, respectively (*P* < 0.001). Chemotherapy failed to decrease the DM rate in the low-risk subclassification, but decreased the DM risk in the intermediate-risk subclassification (*P* = 0.025). In the high-risk subclassificaiton, the DM rate was 31.9% though chemotherapy was used, which was significantly higher than that of other two subclassifications.

Conclusions: DM is the dominant treatment failure in NPC treated by IMRT, with similar occurrence times and distributions to those that occurred in the era of conventional radiotherapy. Further studies on treatment optimization are needed in high-risk patients.

## INTRODUCTION

Nasopharyngeal carcinoma (NPC) is a rare malignancy in most parts of the world, but it is more common in Southeast Asia, particularly among the Southern Chinese population [1]. NPC is highly sensitive to radiotherapy, and intensity-modulated radiotherapy (IMRT) has replaced 2-dimensional radiotherapy (2D-RT) as the first choice for non-disseminated NPC patients. IMRT alone is applied in the treatment of early stage NPC, whereas concurrent chemoradiotherapy (CCRT) with or without neoadjuvant chemotherapy (NACT) or adjuvant chemotherapy (ACT) is recommended for locoregionally advanced NPC according to NCCN guidelines.

With the dose superiority of intensity-modulated radiotherapy (IMRT) technology, excellent locoregional control has been achieved compared with 2D-RT, even in locoregionally advanced patients, with a relatively low incidence of severe complications [2]. However, this improvement in locoregional control has not been accompanied by an increase in long-term overall survival. Reports from other centers have also shown persistently high distant metastasis (DM) rates in patients who received IMRT, resulting in a predominant failure pattern [3–6].

As IMRT has been widely applied around the world, it is necessary to understand the failure patterns, DM features and benefits of chemotherapy in NPC patients who have undergone IMRT in order to provide further guidance regarding treatment choices and clinical trial design.

## RESULTS

### Patient characteristics

The characteristics, treatment factors and treatment parameters of 576 patients are summarized in Table 1. Of these patients, 443 were male, and 133 were female. The median age was 43 years (13-78 years), and 99.8% of the patients were pathologically confirmed to have WHO type IIa or type IIb NPC. All patients received radical IMRT. The average mean dose of GTVnx and GTVnd was 74.3 Gy (63.6-79.8 Gy) and 66.82 Gy (64.83-76.6 Gy), respectively. In total, 376 (65.3%) patients received chemoradiotherapy, and 200 (34.7%) patients were treated with IMRT alone (72 patients were stage III-IV); after 2005, 9 patients with stage III-IV did not receive chemotherapy for the following reasons: age older than 65 years, contraindications to chemotherapy, or patient preference. Among the 376 patients who received chemotherapy, 46 patients had stage II disease, and 328 patients had stage III-IVa-b disease. A total of 236 patients were treated with CCRT alone, 121 patients were treated with NACT followed by CCRT, and 19 patients received other chemotherapy regimens, based on acute toxicity of chemotherapy or their preference [15 patients received NACT alone, 2 patients received CCRT + adjuvant chemotherapy (ACT), and 2 patients received ACT alone]. The ACT schedule was 80 mg/m^2^ cisplatin on day 1 and 72 hours of continuous intravenous infusion of 4.0 g/m^2^ 5-fluorouracil at 3 weeks after IMRT repeated every 3 weeks for two cycles.

**Table 1:** Patient characteristics and treatment factors (*n* = 576)

Characteristics	No.	(%)
Gender		
Male	443	(76.9)
Female	133	(23.1)
Age (years)		
Median (range)	43 (13-78)	
WHO histological type§		
I	1	(0.2)
IIa	56	(9.7)
IIb	519	(90.1)
T-classification*		
T1	96	(16.7)
T2	152	(26.4)
T3	235	(40.8)
T4	93	(16.1)
N-classification*		
N0	78	(13.5)
N1	320	(55.6)
N2	150	(26.0)
N3	28	(4.9)
Clinical stage*		
I	31	(5.4)
II	145	(25.2)
III	283	(49.1)
IVa-b	117	(20.3)
Mean dose to GTVnx (Gy)		
Median (range)	74.29 (63.56-79.81)	
Chemotherapy		
CCRT alone	236	(41.0)
NACT+CCRT	121	(21.0)
CCRT+ACT	2	(0.4)
NACT alone	15	(2.6)
ACT alone	2	(0.4)
No chemotherapy	200	(34.7)

§ According to the 2005 classification.

* According to the UICC/AJCC TNM staging system 7^th^ edition.

### Treatment results and failure patterns

**Figure 1:**
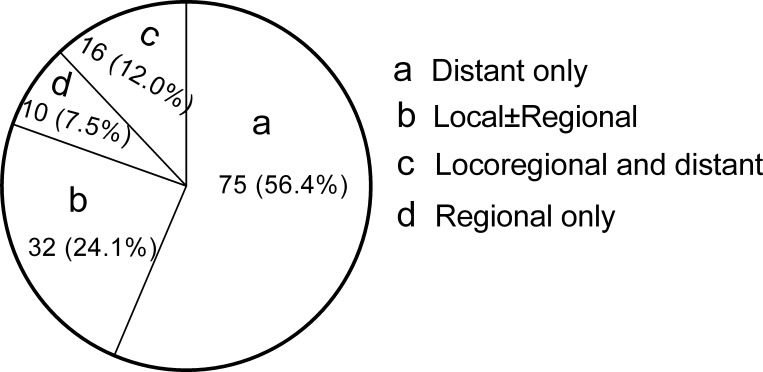
Pie graphs show the treatment failure patterns for the NPC patients who received IMRT

The last follow-up date was December 31, 2014, and the median follow-up time was 103.6 months (range, 4.2-166.8 months) for the entire cohort and 112.3 months (range, 29.4-166.8 months) for the surviving patients. A total of 21 patients (4.0%) were lost to follow-up. At the time of analysis, 133 patients were found to have treatment failures, and 91 patients (68.4%) had at least one site of DM. The 1-, 3-, 5-, and 8-year DM rates were 5.9%, 12.7%, 14.5%, and 16.4%, respectively. Among the failures, 75 experienced DM alone, 42 developed locoregional failure alone, and 16 failed in both distant and locoregional sites (Figure 1). Locoregional control was achieved in most (82.4%) patients who exhibited DM. Specifically, only 5 (10.6%) patients presented failed locoregional control when DMs were diagnosed in the first year after treatment.

### Timing and distribution of DM

The median time interval between treatment and DM detection was 12.2 months (range, 1.3-90.5 months), with 51.6% of the DMs occurring within 1 year after completion of treatment, 72.5% within 2 years, 82.4% within 3 years, 85.7% within 4 years and 92.3% within 5 years. The survival duration after DM ranged from 0.8 to 143.7 months with a median of 14.0 months. The five most common metastatic sites were as follows: bone, 51 cases; lung, 32 cases; liver, 27 cases; distant lymph nodes, 10 cases; and adrenal gland, 2 cases. In total, 68.1% of the patients who experienced DM presented with individual sites. The details are shown in Table 2.

**Table 2:** Metastatic patterns of patients experiencing distant failure after treatment

Metastatic sites	Patients	
	No.	%
Individual sites		
Bone	29	31.9
Lung	17	18.7
Liver	13	14.3
Other sites	3	3.3
Multiple sites With coexisting bone failure		
Bone+liver and/or lung	20	22.0
Bone+other sites	2	2.2
Without coexisting bone failure		
Lung+other sites	3	3.3
Liver+other sites	1	1.1
Other sites	3	3.3
Total	91	100.0

### Hazards according to various T and N subgroups for DM and risk subclassification

**Figure 2:**
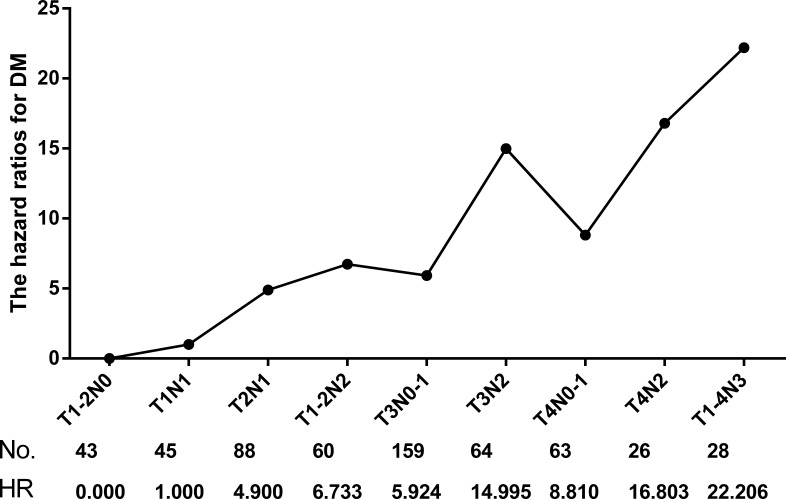
Hazard ratios (HRs) of different T and N combination subgroups for DM

To evaluate the relative risk of DM for different T and N subgroups, the hazard ratios (HRs) for each subgroup were calculated with DM as the endpoint. The HR of patients with T1N1 disease was defined at baseline (HR = 1.00), and host factors (age and sex) and locoregional recurrence were included as covariates. T1N0 was analyzed together with T2N0 due to the limited number of cases. Similarly, T1N2 and T2N2, T3N0 and T3N1, T4N0 and T4N1, and any T with N3 were combined and analyzed. Then, the patients were divided into 9 subgroups. The HR of DM increased with higher T stage and N stage for certain N and T classifications, respectively (Figure 2). Patients with both locally advanced and regionally advanced (T3-4N3) disease had the highest risk for DM (HR = 22.206, *P* = 0.003).

**Figure 3:**
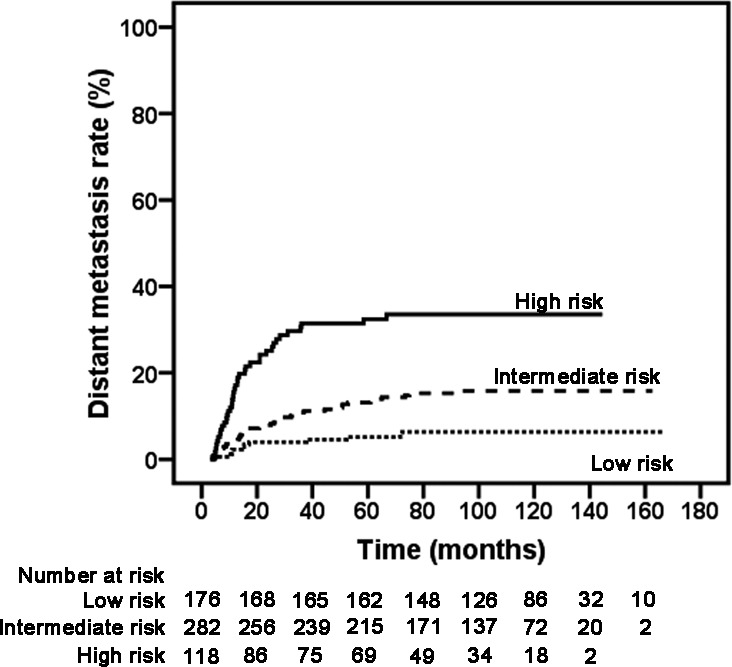
Kaplan-Meier DM rate curves in different risk subclassifications (Low *vs*. Intermediate χ^2^ = 8.646, *P* = 0.003; Low *vs*. High χ^2^ = 38.722, *P* < 0.001; Intermediate *vs*. High χ^2^ = 19.142, *P* < 0.001).

Based on the DM hazard ratio analysis, we were able to classify 576 patients into three subclassifications: low-risk (HR ≤ 5.0), intermediate-risk (HR > 5.0, ≤ 10.0) and high-risk (HR > 10.0). The low-risk subclassification included 176 patients with T1-2N0-1 diseases, wherein only 11 (6.3%) patients had DM. The intermediate-risk subclassification included 282 patients with T3-4N0-1 or T1-2N2 diseases, wherein 42 (14.9%) patients had DM. The high-risk subclassification included 118 patients with T3-4N2 or any T with N3 diseases, wherein 38 (32.2%) patients had DM (Table 3). The 5-year DM rate for low-risk patients was very low at 5.1%, and the rates were 13.1% and 32.4% for intermediate-risk and high-risk patients, respectively. A comparison revealed that the DM rates were significantly different among these three subclassifications (χ^2^ = 43.533, *P* < 0.001) (Figure 3).

**Table 3:** Different risk stratifications and distant failure of nasopharyngeal carcinoma patients treated with IMRT

Risk group	Clinical stage		Patient number	DM	
Low risk	Stage I	T1N0	31	0	
	Stage II	T2N0	14	0	
		T1N1	43	1	
		T2N1	88	10	
	Subtotal		176	11	(6.3%)
Intermediate risk	Stage III	T1N2	17	1	
		T2N2	43	8	
		T3N0	25	2	
		T3N1	134	19	
		T4N0	8	0	
	Stage IVa	T4N1	55	12	
	Subtotal		282	42	(14.9%)
High risk	Stage III	T3N2	64	19	
	Stage IVa	T4N2	26	8	
	Stage IVb	T1-4N3	28	11	
	Subtotal		118	38	(32.2%)

### DM rates of three risk subclassifications treated with chemoradiotherapy

**Figure 4:**
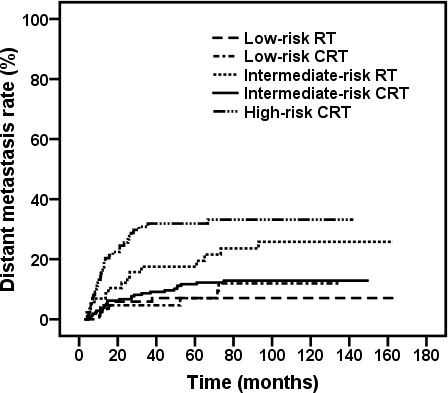
Kaplan-Meier DM rate curves for stage IIb-IVb patients in different risk subclassifications treated with IMRT alone and CCRT±NACT (Low risk: RT *vs*. CCRT, χ^2^ = 0.703, *P* = 0.402; Intermediate risk: RT *vs*. CCRT, χ^2^ = 5.032, *P* = 0.025). In patients who received CCRT ± NACT, low *vs*. intermediate, χ^2^ = 0.054, *P* = 0.816; low *vs*. high, χ^2^ = 7.210, *P* < 0.007; intermediate *vs*. high, χ^2^ = 20.545, *P* < 0.001.

To study the role of CCRT with or without NACT in the different risk subclassifications, we eliminated 17 patients treated with NACT or ACT alone and 2 patients treated with CCRT and ACT. In the low-risk subclassification, 128 patients had stage IIb disease, 85 patients were treated with IMRT alone, and 43 patients received CCRT with or without NACT. The corresponding 5-year DM rates were 7.1% and 7.0% (χ^2^ = 0.703, *P* = 0.402), respectively (Figure 4). In the intermediate-risk subclassification, 58 patients were treated with IMRT alone, and 212 patients received CCRT with or without NACT. The addition of chemotherapy to IMRT was associated with an absolute decrease by 5.8% in the 5-year DM rate for the intermediate-risk subclassification (Figure 4, 17.5% *vs*. 11.7%; χ^2^ = 5.032, *P* = 0.025). In the high-risk subclassification, most patients (100/114) received CCRT with or without NACT, and only 14 were treated with IMRT alone. In patients who received CCRT with or without NACT, the DM rate was significantly higher in high-risk patients than in low-risk patients (Figure 4, 31.9% *vs*. 7.0%, χ^2^ = 7.210, *P* = 0.007) or intermediate-risk patients (Figure 4, 31.9% *vs*. 11.7%, χ^2^ = 20.545, *P* < 0.001). However, no significant difference in the 5-year DM rate was observed between low-risk and intermediate-risk patients treated with CCRT with or without NACT (Figure 4, 7.0% *vs*. 11.7%, χ^2^ = 0.054, *P* = 0.816).

## DISCUSSION

In the past two decades, IMRT has gradually gained popularity in the definitive treatment of NPC. Its technical advantage has already translated into improved clinical outcomes, particularly for the local control rate, which was approximately 90% in our current study and in the literature [3–6, 9]. DM has been the main cause of failure. The 5- and 8-year DM rates of our cohort were 14.5% and 16.4%, respectively.

In previous studies based on 2D-RT, 46.9% to 59.2% of all failure sites after definitive radiotherapy included at least one distant site [10–13], which was lower than the result (68.2%) observed in our study. The main treatment failure patterns in the 2D-RT era included both locoregional recurrence and DM with similar occurrence rates [10, 11, 13, 14]. However, DM alone accounted for 56.1% of all treatment failures and became the predominant pattern, while locoregional failure alone accounted for less than a third of failures. This finding might be attributed to excellent locoregional control after the use of IMRT and advanced diagnostic techniques.

Locoregional failure is a significant adverse prognostic factor for DM in head and neck cancers [15, 16]. Patients with locoregional failures may have more aggressive cancer, which spreads more rapidly, and persistent/recurrent cancer is predisposed to DM. When IMRT was applied, excellent locoregional control was achieved; however, the distant control did not correspond to the decreasing trend of locoregional control. In reality, the DM rate was persistently high. Moreover, 82.4% of DMs were noted in patients with successful locoregional control. A high probability of micrometastases may explain this finding [15, 17, 18]. A portion of patients without obvious clinical evidence of metastases at the time of initial diagnosis may already have had subclinical micrometastases that could not be detected by regular examinations [19], and thus, the increased irradiation dose by IMRT was unable to increase the probability of eliminating micrometastatic lesions. This line of thought is further supported by the early occurrence of DM. In our study, more than half of the DMs occurred within one year, and of these, only 5 (10.6%) patients failed locoregionally when DM was diagnosed.

In the present study, bone was the most common metastatic site and isolated bone metastasis was often observed. Bone was followed by the lung and liver, with other sites rarely appearing. This result is consistent with many other reports, including reports based on 2D-RT [9, 10, 18, 20]. Yi et al. [10] reported a median time of 13 months in a large cohort of NPC patients treated with the 2D-RT technique, which was similar to the median time of 12.2 months after treatment in the present study. The majority (82.4%) of DMs occurred within 3 years of treatment. After 3 years, DM was quite rare. Many other studies from different cancer centers reported similar results [3, 4, 9, 21]. Therefore, close follow-ups 3 years after treatment are necessary to detect distant metastatic lesions and to provide aggressive treatment as early as possible.

The treatment of DM is always a challenge, and the survival time is extremely short despite the use of aggressive treatment [4, 22, 23]. The current study reported a median survival time of 14.4 months after DM, even when individualized therapy was given. Therefore, preventing the development of DM has become a key strategy to improve survival.

The same metastatic timing and distribution between IMRT and 2D-RT likely indicate that IMRT had little effect on distant control, and compared with 2D-RT, the survival benefits of IMRT possibly originated from the high rate of locoregional control. IMRT appears to have a limited contribution to distant control in NPC patients, and new strategies combining different treatment modalities to effectively reduce the rate of DM need to be investigated in the future. In addition, new detection approaches for DM, such as comprehensive pretreatment examinations, including positron emission tomography/computed tomography and tumor markers, may increase the likelihood of detecting metastases.

Based on multiple phase III studies and meta-analyses, concurrent cisplatin-based chemoradiotherapy is the current standard of care for locally advanced disease (AJCCmanual [7th edition] stages II-IVb), as recommended by the latest version of the NCCN [24] and EHNS–ESMO–ESTRO guidelines [25]. Other CRT regimens are also adaptable for locoregionally advanced NPC patients, including NACT+CCRT and CCRT+ACT. However, it is of vital importance to optimize individual treatment strategies for locoregionally advanced patients, particularly considering the various DM risks for different combinations of T and N classifications (Figure 2).

As we classified patients based on the HR of T and N combinations, the 5-year DM rate of the low-risk group was extremely low, regardless of whether the patients received IMRT alone or CRT. For patients with stage IIb disease, it remains uncertain whether CCRT and adjuvant chemotherapy reduced the risk of DM. Despite the survival benefit of CCRT over 2D-RT alone in Mai et al.'s report of a phase III RCT, the results may not be reproducible in the IMRT era. Retrospective data on NPC patients who underwent IMRT in a study by Macao and Tham also did not demonstrate a survival benefit of CCRT in stage IIb patients [26, 27]. Future research may need to focus on improving the quality of life for these patients.

For the intermediate- and high-risk groups, which included patients with locally and/or regionally advanced disease, the 5-year DM rate was 14.1% and 32.7%, respectively. Locoregional treatment alone was therefore inadequate for these two groups of patients. Systemic chemotherapy should be included in the primary treatment for patients with an intermediate or high risk of DM. A meta-analysis with individual data performed by Baujat [28] analyzed 1, 753 patients from eight randomized clinical trials. These authors found that the addition of chemotherapy to standard RT provided a significant survival benefit in patients with NPC, particularly when chemotherapy was administered concomitantly with RT, thus lowering the risk of distant failure (*P* = 0.001; HR, 0.72; 95% CI, 0.59–0.87). However, Lin et al. [29] and Lee et al. [30] reported that chemotherapy failed to improve distant control when IMRT was used for NPC patients. A possible explanation for the discrepancy is the different patient cohorts.

In the present study, the use of chemotherapy benefited the intermediate-risk group with an absolute decline of 6.7% in the 5-year DM rate (*P* = 0.025). Therefore, for the intermediate-risk group, the current treatment regimen of CRT may be used as the standard, and further investigation may be needed to evaluate the utility of NACT or ACT.

In contrast, the DM rate of the high-risk subgroup was 31.9%. Further analysis of the role of chemotherapy in the high-risk group was not performed because of a relatively small number of patients who did not receive chemotherapy. Although aggressive treatments were given to the majority of patients, the outcome was still poor (38/118). A multicenter randomized study in Hong Kong (NPC 0501 Trial) [31] regarding various chemotherapy schemes recently reported its preliminary results that induction cisplatin and capecitabine (PX) presented a favorable trend in progression-free survival compared to adjuvant cisplatin and fluorouracil (PF) (*P* = 0.045). Furthermore, adjusted analyses indicated that induction PX had a lower risk of death (HR, 0.57; 95% CI, 0.34-0.97) than induction PF. Unfortunately, the impact on DM was not analyzed. A more efficacious systemic treatment using more effective regimens and/or different treatment sequences should be included in future clinical trials for high-risk patients. Targeted drugs and immunotherapy also warrant investigation in this subgroup. A closer follow-up after treatment is of vital importance for these NPC patients.

This study was retrospective and has all the limitations of a retrospective analysis. A strength of the study was that it had a large number of patients for characterizing DM.

In conclusion, the current study shows that DM has become the predominant pattern of failure in NPC patients who undergo IMRT with or without chemotherapy. IMRT had no impact on the metastatic timing and distribution of DM. Treatment strategies for different risk stratifications need to be developed. The present study will be useful in guiding future therapeutic trials. More effective systemic chemotherapy and different treatment sequences should be explored for high-risk patients in the future.

## MATERIALS AND METHODS

### Patient selection

Between April 2001 and December 2009, 984 pathologically diagnosed, non-metastatic NPC patients were treated with IMRT with curative intent in our center. To avoid non-uniform treatment strategies used by different oncologists, we enrolled a total of 576 patients who received all of their treatments from our team for this retrospective analysis. The pretreatment workup of all patients included a complete history and physical examination, hematological and biochemical profiles, nasopharyngoscopy, chest radiography, ultrasonography of the abdominal region and whole body emission computed tomography (ECT). Among these patients, 83 (14.4%) underwent head and neck contrast-enhanced computed tomography (CT) scans, and 493 (85.6%) underwent magnetic resonance imaging (MRI) of the head and neck before treatment. For N3 patients or when otherwise indicated, CT scans of the chest and abdomen or positron emission tomography (PET) scans were performed to exclude DM.

### Clinical stage

The patients included were treated from April 2001, and 54 patients were staged according to the 5^th^ edition of the Union for International Cancer Control staging system/American Joint Commission on Cancer (UICC/AJCC) TNM classification. After the publication of the 2002 UICC/AJCC system, the remaining patients were staged according to the 6^th^ edition of the UICC/AJCC TNM classification, and in January 2010, the 7^th^ edition of the UICC/AJCC TNM classification was published and widely used in clinical practice. In the current study, the diseases of all patients were restaged according to the 7^th^ edition of the UICC/AJCC TNM classification based on the recorded clinical and radiological data.

### Patient treatment

All patients received radical IMRT. IMRT was delivered with a dynamic multileaf intensity-modulating collimator (NOMOS Corporation, Sewickley, Pa) using a slice-by-slice arc rotation approach. The details of the IMRT technique and delineation of the target volumes, including the gross tumor volume of the nasopharynx (GTVnx), the positive neck lymph nodes (GTVnd), the high-risk sites of microscopic extension and the whole nasopharynx (CTV1), and the low-risk sites of microscopic extension, the level of the lymph node located, and the elective neck area (CTV2), have been previously described [2, 7]. All organs at risk (OARs), including the brainstem, spinal cord, temporal lobe, optic nerves and chiasm, pituitary, lens, parotid glands, temporomandibular joints, and mandible, were carefully outlined. The prescribed dose was 66-68 Gy to GTVnx, 62-64 Gy to GTVnd, 60 Gy to CTV1 and 54 Gy to CTV2 in 30 fractions. In addition, the prescribed dose for irradiation to the lower neck and the supraclavicular fossae with the conventional RT technique was 50 Gy/25 fractions for prophylactic intent and 60-66 Gy/30-33 fractions for therapeutic intent. The dose constraints to the OARs were reduced as much as possible without sacrificing coverage of the tumor target. The ability to spare these structures depended on the extent and location of the tumor. The maximum doses to these structures were restricted to avoid exceeding their tolerance doses, which were as follows: 56 Gy for the brainstem, 45 Gy for the spinal cord, 60 Gy for the temporal lobes, 8 Gy for the lens, and 60 Gy for the optic nerves and chiasm; the mean doses to the temporomandibular joints, mandible and parotids were 50 Gy, 50 Gy and 40 Gy, respectively.

In our cancer center, chemotherapy was not routinely used for locoregionally advanced NPC until Nov 2004, when Langendijk et al. [8] reported that concurrent chemoradiotherapy (CCRT) was the most effective way to improve overall survival in locoregionally advanced NPC. In our center, cisplatin-based CCRT was administered to patients with stage III-IV disease from 2005 onward. For patients with stage II disease, CCRT was used at the discretion of the attending oncologists, taking into account the patient's preference. Neoadjuvant chemotherapy (NACT) followed by CCRT was administered based on therapeutic clinical trials. The CCRT schedule was IMRT concurrent with intravenous infusion of cisplatin (80 mg/m^2^/day) on days 1 and 22. The NACT schedule was 80 mg/m^2^ cisplatin on day 1 and 4.0 g/m^2^ 5-fluorouracil with 72 hours of continuous intravenous infusion repeated every 3 weeks for two cycles.

For patients developed DM after definitive radiotherapy, chemotherapy is the main treatment choice and usually 4-6 cycles' chemotherapy was given to those patients. The choice of chemotherapy should be individualized based on patients' characteristics, including PS, age, goals of therapy, previous chemotherapy agents and cycles, etc. Generally, combination chemotherapy could be selected for patients in good status and single agents would be preferred for patient in poor condition. Targeted therapy, for specific, cetuximab could also be used in palliative treatment. Local treatments were also given on a case-by-case basis. Positive treatment would be suggested if the patient had solitary metastasis, such as radiation for solitary bone metastasis and ablation for solitary liver metastasis. Palliative adjunctive measures include radiation to areas of symptomatic disease, analgesics, other measures to control other manifestations of disease spread (eg, hypercalcemia) and close monitoring of nutritional status and nutritional support.

### Patient assessment and follow-up

All patients underwent evaluations, including hematological and biochemical profiles and nasopharyngoscopy, at least once a week during treatment. The first assessment of the tumor response was performed by physical examination and nasopharyngoscopy 1 month after the completion of treatment. This assessment was followed by MRI of the head and neck 3 months after radiotherapy. Then, the patients were followed up every 3 months during the first 3 years, every 6 months during years 3-5, and annually thereafter. Each follow-up included complete physical and fiberoptic nasopharyngoscopy or indirect nasopharyngeal speculum examinations. Biochemical profiles, chest X-ray, ultrasound of the liver and abdomen and MRI of the head and neck were also routine elements of the assessment. Further investigations were performed when clinically indicated. Local recurrence was confirmed by biopsy for most patients. Patients with recurrence in inaccessible sites, such as the skull base and intracranial cavity, were diagnosed radiologically according to local disease progression. DM was defined as having one or more of the following conditions: (1) histologically confirmed DM; (2) equivocal evidence of DM in the imaging study and subsequent histological evidence or clinical progression; and (3) unfeasible biopsy of the lesion of interest (e.g., bone site), but with the presence of DM confirmed by two types of imaging studies (e.g., MRI and ECT/or PET) with a concordant clinical course.

### Statistical methods

DM was assessed as the endpoint. The time periods were calculated from the date of pathological diagnosis to the date of DM diagnosis. The DM rate was calculated with the Kaplan-Meier method, and the differences were compared with a log-rank test. Hazard ratios were calculated by a Cox proportion hazards model (backward Wald). Two-tailed values of *P* < 0.05 were considered significant. Statistical analysis was performed using the SPSS software package (Version 16.0, SPSS Inc., Chicago, IL).
